# Diversity Patterns of Bermuda Grass along Latitudinal Gradients at Different Temperatures in Southeastern China

**DOI:** 10.3390/plants9121778

**Published:** 2020-12-15

**Authors:** Jing-Xue Zhang, Ming-Hui Chen, Lu Gan, Chuan-Jie Zhang, Yu Shen, Jin Qian, Meng-Li Han, Yu-Xia Guo, Xue-Bing Yan

**Affiliations:** 1 College of Animal Science and Technology, Yangzhou University, Yangzhou 225000, China; dx120190128@yzu.edu.cn (J.-X.Z.); dx120170094@yzu.edu.cn (M.-H.C.); ganlu2019@yzu.edu.cn (L.G.); chuanjiezhang@yzu.edu.cn (C.-J.Z.); mz120170925@yzu.edu.cn (Y.S.); mz120170924@yzu.edu.cn (J.Q.); MX120190685@yzu.edu.cn (M.-L.H.); 2 College of Animal and Veterinary Science, Henan Agricultural University, Zhengzhou 450002, China

**Keywords:** *Cynodon*, evolution, latitude, SNP, temperature

## Abstract

*Cynodon dactylon* (L.) Pers. (common Bermuda grass) has a limited capacity to grow at low temperatures, which limits its geographical range. Exploring its evolutionary relationship across different environmental gradients is necessary to understand the effects of temperature change on the genetics of common Bermuda grass. In this study, high-throughput transcriptome sequencing was performed on 137 samples of *C. dactylon* from 16 latitudinal gradients to explore the differential molecular markers and analyze genetic diversity and structure along latitudinal gradients at different temperatures. We primarily sampled more high-quality single nucleotide polymorphisms (SNPs) from populations at lower and middle latitudes. Greater intraspecific genetic variation at each level of temperature treatment could be due to factors such as wind pollination and asexual breeding. Populations of *C. dactylon* at high latitudes differed from populations at middle and low latitudes, which was supported by a principal component analysis (PCA) and genetic structure analysis, performed at different temperatures. We observed more genetic variation for low-latitude populations at 5 °C, according to an analysis of three phylogenetic trees at different temperature levels, suggesting that low temperatures affected samples with low cold resistance. Based on the results of phylogenetic analysis, we found that samples from high latitudes evolved earlier than most samples at low latitudes. The results provide a comprehensive understanding of the evolutionary phenomenon of landscape genetics, laying the groundwork for future structural and comparative genomic studies of *C. dactylon*.

## 1. Introduction

A major focus of evolutionary biology is understanding how genetic variation drives local adaptions across populations spanning wide geographical and environmental gradients. While genetic mutations and genetic drift tend to induce divergence in plant populations, natural selection produces similar adaptations to similar environments. It is important to characterize relative genotypic differences across different locations based on multiple environmental tests [1]. Natural genetic differences related to the drought stress tolerance of plants, such as *Festuca arundinacea* Schreb and *Lolium perenne* L., have been studied [2,3]. Adaptations to this kind of stress have important implications for plant growth and adaptation to climate change [4,5]. *C. dactylon* is a warm-season turfgrass primarily ranging from latitudes of 45° N to 45° S. It is widely used for lawns, parks, and sports fields [6]. *C. dactylon* is an ideal species for studying genetic and genomic evolution because of its extraordinary morphological diversity and ecological adaptations. Additional studies are needed to better understand the ability of populations to adapt to global climatic changes that are altering local environmental conditions [7]. Temperature has a major effect on the geographic distribution of plants [8]. Warm-season turfgrass with a C4 photosynthetic pathway grows well between temperatures of 24 °C and 29 °C [9]. *C. dactylon* is more sensitive to cold stress than heat stress [10,11]. When mean temperatures decline to 15 °C, the growth of development of *C. dactylon* slows, while leaf senescence occurs at 10 °C [12]. The response of common Bermuda grass accessions to cold temperatures varies greatly. The varying responses of the morphological structure and function of several differentially expressed proteins in *C. dactylon* to low temperatures have been studied [13]. There is considerable genetic variation in how natural populations of *C. dactylon* tolerate various environmental stresses [10]. As such, this study examines genetic variation in natural populations of *Cynodon dactylon* (L.) Pers. (common Bermuda grass) with a wide distribution at different temperatures.

Genetic divergence among individuals within a population can lead to population divergence through adaptive selection responding differently to environmental factors. Additionally, populations could converge if similar genetic variants in different populations are selected. Transcriptomics can help identify climatic factors affecting plant traits and performance. As more plant genomes and transcriptomes are sequenced, further exploration of the causes and effects of evolution becomes possible. Transcriptome sequencing has resulted in a better understanding of genetic and genomic diversification in non-model plants [14,15,16,17]. It is also a powerful tool for developing useful SNPs throughout the genome [18,19]. Single nucleotide polymorphisms (SNPs) have emerged as a useful molecular markers for analyzing genetic variation within populations [20,21,22]. The genetic diversity analysis of *Opisthopappus Shih* populations by single nucleotide polymorphisms (SNP) was performed in order to obtain valuable information about population genetics [23]. The transcriptomes of two wild populations of *C. dactylon* with different directions of stem growth was studied to better understand genetic diversification [24]. There is limited information about the effects of different environments along latitudinal gradients on population structure, based on SNPs obtained via transcriptome sequencing. The genetic differences along latitudinal gradients at different temperatures could be used to predict genetic features of past environments and the ability to adapt to environmental changes in the future. Landscape genetics studies the ability to produce and transmit adaptive genes [25]. Studying genetic diversity and population structures in different environments helps to identify admixture events, which indicate their potential to adapt to new environmental conditions [26,27]. In this study, we obtained SNPs via transcriptome sequencing and analyzed how genetic variation and structure in 16 populations of Bermuda grass from different latitudinal gradients respond to three different levels of temperature, helping us to better understand plant diversity and evolution along latitudinal gradients and providing a molecular genetic basis for the conservation of natural genetic diversity. It will also contribute to efforts to breed increasingly cold-resistant Bermuda grass.

## 2. Results

### 2.1. Genetic Variation of 16 Populations of Cynodon along Latitudinal at Different Temperatures

A total of 20,684,192 high-quality SNPs were selected from the 553,110 unigenes from the *C. dactylon* samples to further analyze their population genetics. Considerable genetic variation was estimated among populations (Table 1). Nei’s diversity index (Nei’s) and Shannon’s index (I) were computed for each population at different temperatures based on the obtained allele frequencies. Polymorphism information content (PIC) values were calculated using the formula [28] (Equation (1)):

(1) $$\mathrm{PIC}=1-\sum_{j=1}^{n}p_{\mathrm{ij}}^{2}$$

where P_ij_ is the frequency of jth allele for ith locus and summation extends over n alleles.

Overall values were, Nei’s = 0.357, 0.353, 0.360, Shanon Wiener index (I) = 0.526, 0.521, 0.529 and polymorphism information content (PIC) = 0.280, 0.277, 0.282 at 5 °C, 20 °C and 35 °C, respectively. Observed heterozygosity (HO) estimates among all populations and expected heterozygosity (HE) were also calculated to indicate genetic diversity varied among populations. Diversity estimates for some populations at high latitude were a little lower than for the others.

### 2.2. Phylogenetic Relationship along Latitudinal Gradients at Different Temperatures

**Figure 1:**
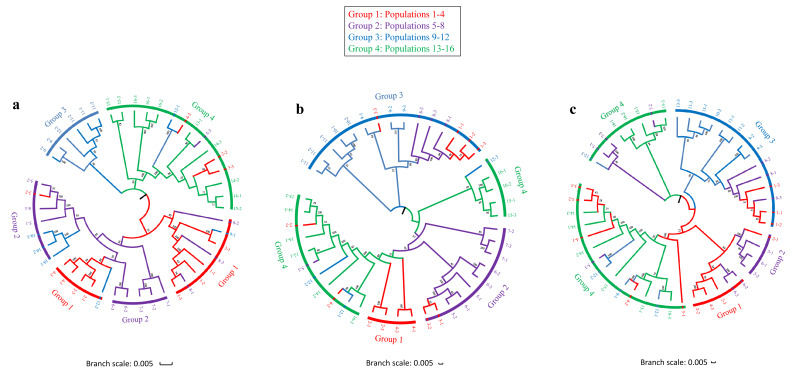
Maximum-likelihood phylogeny of 16 populations of *C. dactylon*. (**a**) phylogeny of *C. dactylon* at 5 °C. (**b**) phylogeny of *C. dactylon* at 20 °C. (**c**) phylogeny of *C. dactylon* at 35 °C. Group 1 contains populations 1–4; Group 2 contains populations 5–8; Group 3 contains populations 9–12; Group 4 contains populations 13–16.

We analyzed the relationships between different populations along latitudinal gradients using SNP data at different temperature levels by constructing three trees for each temperature treatment (Figure 1). The phylogenetic tree at 20 °C clearly separated the mid-latitude populations (Group 3) from the other populations. Individuals at 5 °C formed two clades, composed of populations at low latitudes (Group 1 and Group 2) and high latitudes (Group 3 and Group 4). Compared to the tree at 20 °C, individuals from Group 1 and Group 2 clustered with individuals at 5 °C, which indicates there could be genetic variation in low-latitude populations. The phylogenetic tree of individuals at 35 °C showed almost the same four clades as at 20 °C. However, we also observed inconsistent patterns between these two trees. Some individuals at high latitudes (populations 7, 15, 16) clustered together, while some individuals from the other populations were clustered at 35 °C. Additionally, under three temperature treatments, population one clustered together with mid-latitude populations (populations 8–10). We observed widespread admixture across different populations of *C. dactylon*, which could be due to the gene introgression of different genetic populations, particularly those that shared similar geographic origins. Individuals at high latitudes evolved earlier than most individuals at low latitudes. No obvious linear trend of genetic similarity was observed among populations from low to high latitudes, suggesting a divergent pattern of evolution in *C. dactylon* along latitudinal boundaries. We also observed frequent introgressive hybridization in *C. dactylon* along latitudinal gradients.

### 2.3. Population Genetic Structure

After additional analysis of molecular variance (AMOVA) analyses, we found that more variation occurred at a temperature of 5 °C (SS = 91,708.467) and 35 °C (SS = 90,139.609), compared to 20 °C (SS = 57,584.466). High genetic differentiation was detected within populations at different temperature levels. Additionally, lower genetic differentiation was observed among populations from all latitudes when grown in low and high temperatures than when grown at 20 °C, whereas relatively low genetic differentiation was observed within populations at 20 °C (Table 2).

**Figure 2:**
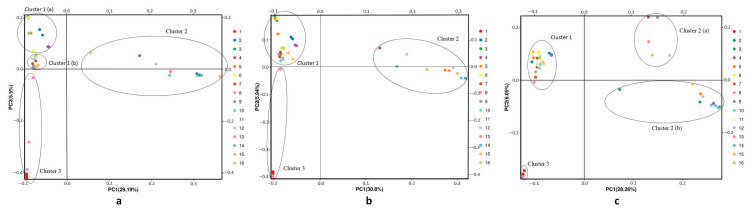
Principal component analysis (PCA) of 16 *C. dactylon* populations at different temperatures. Different colors indicate different populations. Black circles represent genetic clusters. (**a**) PCA of *C. dactylon* at 5 °C. (**b**) PCA of *C. dactylon* at 20 °C. (**c**) PCA of *C. dactylon* at 35 °C.

SNPs based on the *C. dactylon* transcriptome sequence (20,684,192 SNPs) were used to investigate genetic structure at different temperatures using principal component analysis (PCA) (Figure 2). The PC1 accounted for 29.19%, 30.80%, and 28.26% of the variance in SNP data at 5 °C, 20 °C, and 35 °C, respectively, while the PC2 accounted for 5.50%, 5.94%, and 6.05% of the variance in SNP data at 5 °C, 20 °C, and 35 °C, respectively. The PCA plot of *C. dactylon* populations at 20 °C shows that most individuals at low latitudes and middle latitudes were grouped into cluster one, while two populations (number one and eight) were grouped in cluster three. The high-latitude populations grouped in cluster two were clearly separated from the others. The principal component analysis (PCA) (Figure 2) revealed a similar pattern, as indicated by the UPGMA (Unweighted Pair-group Method with Arithmetic Mean) dendrogram. In the PCA plot of *C. dactylon* populations at 35 °C, on axis one, the 16 populations were divided into two parts: one consisting of cluster two and the other of clusters one and three. On axis two, cluster two consists of high-latitude populations that are divided into cluster two (a) and cluster two (b). However, at axis two, cluster one (a) consists of low-latitude populations that could be separated from cluster one (b) based on the results of PCoA on *C. dactylon* populations at 5 °C.

**Figure 3:**
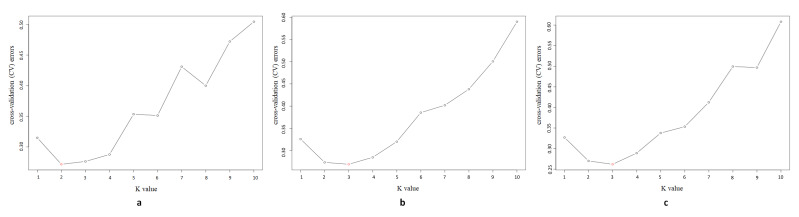
Cross-validation (CV) errors of different K values (k = 1–10) at different temperatures. Red circles represent CV errors of best K-value. (**a**) CV errors at 5 °C. (**b**) CV errors at 20 °C. (**c**) CV errors at 35 °C.

**Figure 4:**
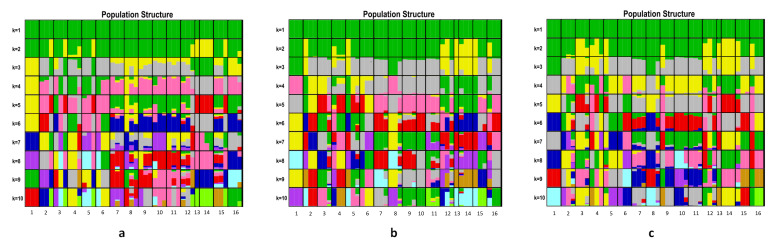
Genetic structure 16 populations of *C. dactylon* along latitudinal gradient at different temperatures. The STRUCTURE ancestry kinship (K) is shown from 1 to 10. Various colors indicate different genetic clusters. (**a**) structure of *C. dactylon* at 5 °C. (**b**) structure of *C. dactylon* at 20 °C. (**c**) structure of *C. dactylon* at 35 °C.

The genetic structure coincided with the clustered results of *C. dactylon* (K) from one to 10. The best K-value along latitudinal gradients for our analysis was estimated as K = 2, K = 3 and K = 3 at 5 °C, 20 °C, and 35 °C, respectively (Figure 3). In the structure analysis of the *C. dactylon* populations at three different temperatures, most high-latitude samples differed from the other populations at middle latitudes and low latitudes (Figure 4). When K = 2, based on the results of structure on *C. dactylon* populations at 5 °C, most high-latitude samples remained in the yellow cluster. Additionally, most samples at middle latitudes and low latitudes remained in the green cluster. When K = 3, most samples at middle latitudes and low latitudes remained in the gray cluster according to the results of structure on *C. dactylon* populations at 20 °C and 35 °C, while most high-latitude samples remained in the yellow cluster at 20 °C and 35 °C. Some of these mid-latitude individuals had close evolutionary relationships with low-latitude individuals. Population one was different from low-latitude populations, and grouped together with population eight. When K = 4, there were four clusters at different temperatures: populations at low-latitude, mid-latitude, high-latitude, population one and eight clustered together.

## 3. Discussion

### 3.1. Population Genetic Differentiation and Structure of C. dactylon

The relationship between local selection and adaptation with climate variables across different geographic locations could be associated with longstanding evolutionary mechanisms. Local selection and genetic drift could shape genetic diversity and differentiation patterns for *C. dactylon* along a latitudinal gradient. Greater intraspecific genetic variation, based on our analysis of molecular variance (AMOVA), could confer a competitive advantage in a changing environment. These results agree with the published results of our analysis of the genetic structure using expressed sequence tag-derived simple sequence repeats (EST-SSR) [29]. Higher environmental heterogeneity could favor higher tolerance of environmental factors and a broader range of acclimation responses [30,31]. This could explain the genetically diverse populations we identified when analyzing genetic structures. Populations of *C. dactylon* at high latitudes differed from other populations at middle latitudes and lower latitudes based on an analysis of their genetic structure. The results of both the genetic structure analysis and the PCA analysis corresponded to growth habits [32,33,34]. Migration among different populations was assumed in our structure analyses, while gene flow could have caused populations at different latitudes to adapt to similar conditions, which has been well-researched [29]. Proposed adaptive introgression could allow for adaptation and establishment in different environmental conditions [35]. Some of these individuals were admixed, which indicates that hybridization and introgression occurred during the evolutionary process.

Additionally, *C. dactylon* at low latitudes could emerge directly from most individuals at high latitudes, according to the results of our phylogenetic analysis at different temperatures. Most individuals at low latitudes are descendants of populations located at high latitudes, which could be due to the evolution of genetic diversity along latitudinal gradients. Biogeographic studies have demonstrated a southward migration towards warmer regions [36,37]. Our results also indicate that phylogenetic clustering at high latitudes, along with the phylogenetic structure, tended to disperse individuals at low latitudes, due to favorable temperatures. The evolution of plants, along with geological events, could help understand evolutionary history. Climatic fluctuations and numerous, complex ecological niches could promote north–south exchanges [38]. Phylogeographic studies and additional investigation into climatic and environmental changes provide a new perspective on the history of population changes.

### 3.2. Analysis of C. dactylon Landscape Genetics

These results demonstrate the importance of identifying the geographical source of a sample during an experiment, as well as the need to study different temperature levels in order to fully understand how genetic variation affects the adaptive abilities of *C. dactylon*. Genetic resources of wild plant species are helpful for plant breeding [39,40], so the germplasm from wild Bermuda grass collected from all over the world is significant for the conservation of natural genetic diversity. The rapid development of high-throughput molecular methods (such as SNPs) used in this study has allowed us to better understand the differences and evolutionary patterns of *C. dactylon*. Individuals at low latitudes have likely adapted to relatively higher temperatures, while low temperatures affected the genetic variation of populations at low latitudes. Across evolutionary time, individuals typically appeared at low latitudes subsequent to appearing at high latitudes, indicating that *C. dactylon* could have evolved from northern latitudes to southern latitudes. An analysis of genetic structure determined that populations of *C. dactylon* at high latitudes differed from populations at middle latitudes and low latitudes. Understanding genetic variation across geographic boundaries provides a deeper understanding of the study of evolutionary patterns. Our study suggests a valuable pattern of population genetics at different latitudes for the genomic evolution of *C. dactylon*. Additionally, some populations and individuals can be used to breed strains that are highly resistant to cold temperatures. This analysis will provide the foundation for future structural and comparative genomic studies of *C. dactylon*, and will also provide additional insight into the evolutionary phenomenon of landscape genetics.

### 3.3. Adaptation of Genetic Variation along Latitudinal Gradients to Different Temperatures

Genetic variation among populations can occur as an adaptation to a wide range of climatic conditions, including temperature [41,42]. Inverse relationships between latitude and diversification rate have been observed; temperature, in particular, has the highest impact on the diversification rate of plants [43,44]. In our study, higher levels of genetic differentiation within populations were observed at 5 °C than at 20 °C. Additionally, samples from low latitudes were clearly separated from samples from high-latitudes at 5 °C. Temperature is one of the primary drivers of community change in plants [45]. This may indicate that low temperatures affected the genetic variation of populations at low latitudes, which had low resistance to cold temperatures. Guangzhou (population two), Yingde (population three), Renhua (population four), Guidong (population five), Youxian (population six), and Liuyang (population seven) are all located at low latitudes and have high annual average temperatures. Low temperatures limit the distribution and development of most plant species [13]. For example, southern lineages of scallops located at higher latitudes live in warmer offshore waters because they have adapted to high temperatures [46]. Genetic variation should allow populations of *A. millepora* to adapt to gradual warming, while novel genetic mutations could induce further adaptations [47]. High temperatures could affect populations from Cixian (population 16) and Huixian (population 15), which are located at relatively high latitudes. Higher temperatures are correlated with high rates of genetic mutation, based on the evolutionary speed hypothesis [48]. Additionally, populations from Liuyang (population 7) could be affected by both low and high temperatures. Populations at different latitudes could adapt differently to different temperatures due to genetic diversity among populations. Local adaptation to different temperature conditions promotes evolutionary diversification [49]. Increased heterozygosity of polyploids at low and high latitudes helps to adapt to different environmental conditions [50]. To make the results more comprehensive, we identified differentially expressed genes (DEGs) that were highly correlated with the diversification of *C. dactylon* for functional analysis associated with temperature (unpublished paper).

## 4. Material and Methods

### 4.1. Plant Material and Experimental Design

**Figure 5:**
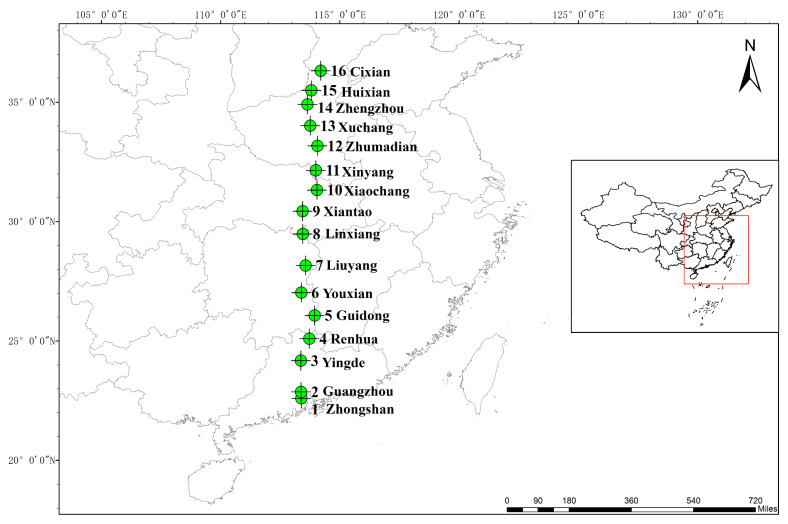
Distribution of *C. dactylon* populations in southeastern China. The green dot represents the sampling sites of transcriptome sequencing.

All 137 samples of *Cynodon dactylon* (L.) Pers. were collected from 16 geographic sites across China, ranging from 22°35′ N to 36°18′ N (Figure 5; Table S1). They were then all planted in the experimental farm of Yangzhou University, Yangzhou, China, in 2018. We sampled 48 individuals (with random three individuals from 20 individuals for each population) and planted them at three different temperatures: 5 °C, 20 °C and 35 °C for 2 months. The growing conditions were the same within the three climatic chambers: the humidity was 60%, the photoperiod was 12 h d^−1^, and the luminosity was 600 μmol photon m^−2^s^−1^. Field experiments were based on a randomized complete block design within each climatic chamber. Forty-eight individuals of *C. dactylon* from the 16 populations were cultivated on a plot, while the distance between the plants was 10 cm and the distance between rows was 20 cm. The leaves of each plant in each growing chamber were sampled and immediately flash frozen in liquid nitrogen for RNA extraction.

### 4.2. RNA Extraction and Sequencing

RNA was extracted using the Plant RNA Kit (OMEGA, Norcross, GA, USA) according to the manufacturer’s instructions. The RNA concentration and quality were determined using a NanoDrop spectrophotometer (Thermo Scientific, Wilmington, DE, USA) and an Agilent Bioanalyzer (Agilent, Santa Clara, CA, USA). High-quality mRNA obtained from the total RNA sample and oligo dT primer was then used for reverse transcription (RT) to generate cDNA, using a cDNA synthesis kit (Clontech Laboratories, Mountain View, CA, USA). All of the products of PCR reactions following RT were purified using AMPure PB beads (Pacific Biosciences, Menlo Park, CA, USA), while the DNA fragments were selected using the BluePippin System. The products were then quantified using an Agilent 2100 Bioanalyzer (Agilent Technologies, Inc., Santa Clara, CA, USA), while the sequencing libraries were prepared using Illumina TruSeq kits. Paired-end sequencing with an insert size of ∼150 bp was performed using the Illumina HiSeq 2000 platform. Clean reads were obtained using a stringent filtering process and an adapter.

### 4.3. Detection of SNP Population and Data Analysis

SNPs for each individual were analyzed using the GATK (The Genome Analysis Toolkit, Broad Institute) software [51]. From transcriptome sequence of non-model plant, we obtained high-quality SNPs (Table S2) and excluded SNP calling errors. We analyzed the values of genetic variation among and within populations using the AMOVA analysis, while the significance was tested using 999 permutations generated by the ARLEQUIN software [52]. We also used principal component analysis (PCA) using the EIGENSOFT software to quantify the distribution of genetic variation at different latitudes [53].

### 4.4. Population Structure and Phylogenetic Tree

The population structure at different latitudes was analyzed using the ADMIXTURE software, with a burn-in period of 10,000 generations and 50,000 iterations [54]. We tested populations (K) from 1 to 10 with default convergence criterion, while the best K-value was selected based on the lowest value of cross-validation errors (CV). Individuals along a latitudinal gradient were separated into four groups with four populations of one group by latitude from south to north. A tree illustrating the genetic relationship among the four groups at different temperatures was generated based on SNP data using MEGAX (MEGA-CC) and the Kimura 2-parameter model with 1000 bootstrap replicates.

## Figures and Tables

**Table 1:** Genetic diversity indexes of *C. dactylon* of different population at different temperatures.

	5 °C					20 °C					35 °C				
	Ho	He	Nei’s	I	PIC	Ho	He	Nei’s	I	PIC	Ho	He	Nei’s	I	PIC
**1**	0.914	0.479	0.574	0.670	0.362	0.893	0.475	0.570	0.666	0.360	0.911	0.478	0.574	0.670	0.362
**2**	0.700	0.418	0.501	0.604	0.326	0.702	0.416	0.499	0.602	0.324	0.730	0.432	0.519	0.620	0.335
**3**	0.673	0.410	0.492	0.596	0.321	0.779	0.448	0.537	0.637	0.344	0.788	0.445	0.534	0.634	0.343
**4**	0.726	0.427	0.512	0.614	0.331	0.713	0.425	0.510	0.612	0.330	0.699	0.423	0.508	0.611	0.329
**5**	0.687	0.416	0.499	0.602	0.325	0.700	0.416	0.499	0.602	0.325	0.676	0.410	0.492	0.596	0.321
**6**	0.801	0.450	0.540	0.640	0.346	0.750	0.442	0.530	0.631	0.341	0.769	0.445	0.534	0.634	0.342
**7**	0.780	0.448	0.537	0.637	0.344	0.835	0.459	0.551	0.649	0.351	0.791	0.446	0.535	0.635	0.343
**8**	0.791	0.448	0.538	0.638	0.345	0.816	0.456	0.548	0.647	0.350	0.840	0.461	0.553	0.651	0.352
**9**	0.783	0.447	0.537	0.636	0.344	0.771	0.445	0.534	0.634	0.343	0.714	0.421	0.505	0.607	0.327
**10**	0.837	0.458	0.549	0.648	0.350	0.915	0.480	0.640	0.672	0.364	0.771	0.446	0.535	0.635	0.343
**11**	0.784	0.447	0.537	0.636	0.344	0.709	0.432	0.518	0.620	0.335	0.776	0.444	0.533	0.633	0.342
**12**	0.683	0.414	0.497	0.600	0.324	0.726	0.430	0.516	0.618	0.334	0.691	0.425	0.510	0.613	0.331
**14**	0.815	0.455	0.546	0.645	0.348	0.831	0.458	0.550	0.648	0.350	0.865	0.467	0.560	0.657	0.355
**15**	0.683	0.414	0.496	0.600	0.323	0.618	0.398	0.478	0.583	0.314	0.653	0.406	0.487	0.591	0.319
**16**	0.820	0.449	0.539	0.638	0.345	0.711	0.423	0.508	0.610	0.329	0.695	0.425	0.510	0.612	0.330
**Overall**	0.543	0.353	0.357	0.526	0.280	0.528	0.349	0.353	0.521	0.277	0.539	0.356	0.360	0.529	0.282

Ho, observed heterozygosity; He, expected heterozygosity; Nei’s, Nei diversity index; I, Shanon Wiener index; PIC, polymorphism information content.

**Table 2:** Analysis of molecular variance (AMOVA) of *C. dactylon* of different population at different temperatures.

**5 °C**						
**Source of Variation**	**df**	**SS**	**MS**	**Estimate of Variation**	**%**	**Fixation Index (Fst)**
**Among Populations**	15	44,715.134	2981.008933	4.11	5.56%	0.041 *
**Within Populations**	30	46,993.333	1566.444433	69.78	94.44%	
**Total**	45	91,708.467		73.89		
**20 °C**						
**Source of Variation**	**df**	**SS**	**MS**	**Estimate of Variation**	**%**	**Fixation Index (Fst)**
**Among Populations**	15	28,819.383	1921.2922	4.36	5.90%	0.044 *
**Within populations**	29	28,765.083	991.8994138	69.5	94.10%	
**Total**	44	57,584.466		73.86		
**35 °C**						
**Source of Variation**	**df**	**SS**	**MS**	**Estimate of Variation**	**%**	**Fixation Index (Fst)**
**Among Populations**	15	43,718.942	2914.596133	4.16	5.70%	0.042 *
**Within Populations**	30	46,420.667	1547.355567	68.85	94.30%	
**Total**	45	90,139.609		73.01		

Note: df, degree of freedom, SS, sum of squares, MS, mean of squares; *, significant at 5% level (*p* < 0.05).

## Supplementary Materials

The following are available online at https://www.mdpi.com/2223-7747/9/12/1778/s1, Table S1: Geographical distribution of 16 *C. dactylon* sampled populations. Table S2: SNP numbers of *C. dactylon* among latitudinal gradient at different temperature.
