# Supplementary material for: Diversity Patterns of Bermuda Grass along Latitudinal Gradients at Different Temperatures in Southeastern China
Source: Plants (Basel). 2020 Dec 15;9(12):1778. doi: 10.3390/plants9121778 (PMC7765174; doi:10.3390/plants9121778)
Supplement: Supplementary file 1 [file plants-09-01778-s001.pdf]

**Supplementary Tables:**

**Table S1.** Geographical distribution of 16 *C.dactylon* sampled populations.

| Population code | Location | Latitude | Longitude | Altitude(m) | Habitat | Annual average temperature(℃) |
|---|---|---|---|---|---|---|
| 1 | Zhongshan | 22°35′40″ | 113°23′17″ | 0 | Roadside | 22.0 |
| 2 | Guangzhou | 22°51′48″ | 113°22′22″ | 10 | Roadside | 22.8 |
| 3 | Yingde | 24°10′31″ | 113°22′08″ | 50 | Roadside | 21.2 |
| 4 | Renhua | 25°05′29″ | 113°43′17″ | 90 | Roadside | 19.9 |
| 5 | Guidong | 26°03′49″ | 113°56′34″ | 810 | Slope rangeland | 15.8 |
| 6 | Youxian | 27°00′59″ | 113°23′07″ | 90 | Roadside | 18.1 |
| 7 | Liuyang | 28°09′14″ | 113°33′42″ | 90 | Roadside | 17.5 |
| 8 | Linxiang | 29°28′32″ | 113°26′48″ | 60 | Roadside | 16.8 |
| 9 | Xiantao | 30°25′48″ | 113°26′05″ | 30 | Roadside | 17.0 |
| 10 | Xiaochang | 31°18′59″ | 114°02′15″ | 50 | Roadside | 16.8 |
| 11 | Xinyang | 32°08′38″ | 113°59′46″ | 100 | Roadside | 15.5 |
| 12 | Zhumadian | 33°09′47″ | 114°03′45″ | 50 | Arable land | 15.2 |
| 13 | Xuchang | 34°00′30″ | 113°45′23″ | 90 | Roadside | 14.6 |
| 14 | Zhengzhou | 34°54′04″ | 113°38′20″ | 90 | Roadside | 14.7 |
| 15 | Huixian | 35°29′26″ | 113°48′23″ | 120 | Roadside | 14.6 |
| 16 | Cixian | 36°18′40″ | 114°11′51″ | 130 | Roadside | 13.4 |

**Table S2.** SNP numbers of *C. dactylon* among latitudinal gradient at different temperature.

| **Population Code** | **5 ℃** | | | **20 ℃** | | | **35 ℃** | | |
|---|---|---|---|---|---|---|---|---|---|
| | **HomoSNP** | **HeteSNP** | **All SNP** | **HomoSNP** | **HeteSNP** | **All SNP** | **HomoSNP** | **HeteSNP** | **All SNP** |
| 1 | 439527 | 113528 | 553055 | 320597 | 81358 | 401955 | 417792 | 109573 | 527365 |
| 2 | 370806 | 113595 | 484401 | 348221 | 113825 | 462046 | 366858 | 111166 | 478024 |
| 3 | 378700 | 111016 | 489716 | 291681 | 72184 | 363865 | 372699 | 123598 | 496297 |
| 4 | 327824 | 115300 | 443124 | 277479 | 91078 | 368557 | 350772 | 119368 | 470140 |
| 5 | 375878 | 100595 | 476473 | 332341 | 95595 | 427936 | 347171 | 102072 | 449243 |
| 6 | 402602 | 105948 | 508550 | 251247 | 60001 | 311248 | 365925 | 91344 | 457269 |
| 7 | 359554 | 92373 | 451927 | 370182 | 98894 | 469076 | 412980 | 114999 | 527979 |
| 8 | 394219 | 103848 | 498067 | 327534 | 82078 | 409612 | 452143 | 124713 | 576856 |
| 9 | 386022 | 97276 | 483298 | 304239 | 76303 | 380542 | 396721 | 115171 | 511892 |
| 10 | 376457 | 101774 | 478231 | 199825 | 55516 | 255341 | 313755 | 80033 | 393788 |
| 11 | 354887 | 88430 | 443317 | 216276 | 51655 | 267931 | 385425 | 102566 | 487991 |
| 12 | 359411 | 103132 | 462543 | 315066 | 106543 | 421609 | 353801 | 108416 | 462217 |
| 13 | 94541 | 30506 | 125047 | 106396 | 37024 | 143420 | 93443 | 31222 | 124665 |
| 14 | 358083 | 120526 | 478609 | 363796 | 133344 | 497140 | 377139 | 130029 | 507168 |
| 15 | 405129 | 116545 | 521674 | 225913 | 59714 | 285627 | 300909 | 84388 | 385297 |
| 16 | 400994 | 110121 | 511115 | 356900 | 111968 | 468868 | 374300 | 109781 | 484081 |
| Total | 5784634 | 1624513 | 7409147 | 4607693 | 1327080 | 5934773 | 5681833 | 1658439 | 7340272 |
